# From Optical Tracking to Tactical Performance via Voronoi Diagrams: Team Formation and Players’ Roles Constrain Interpersonal Linkages in High-Level Football

**DOI:** 10.3390/s23010273

**Published:** 2022-12-27

**Authors:** Nelson Caldeira, Rui J. Lopes, Dinis Fernandes, Duarte Araujo

**Affiliations:** 1 CIPER, Faculty of Human Kinetics, University of Lisbon, 1499-002 Cruz Quebrada, Portugal; 2 Department of Information Science and Technology (ISTA), Iscte—Instituto Universitário de Lisboa, 1649-026 Lisbon, Portugal; 3 Instituto de Telecomunicações, 1049-001 Lisbon, Portugal

**Keywords:** affordances, spatial dominance patterns, performance, team synergies, Voronoi cells

## Abstract

Football performance behaviour relies on the individual and collective perceptual attunement to the opportunities for action (affordances) available in a given competitive environment. Such perception–action coupling is constrained by players’ spatial dominance. Aiming to understand the influence of team formation and players’ roles in their dynamic interaction (interpersonal linkages), Voronoi diagrams were used to assess the differences in players’ spatial dominance resulting from their interactions according to ball-possession status in high-performance football. Notational (i.e., team formation, players’ role, and ball-possession status) and positional data (from optical sensors) from ten matches of the men’s French main football league were analysed. Voronoi diagrams were computed from players’ positional data for both teams. Probability density functions of the players’ Voronoi cell areas were then computed and compared, using the Kolmogorov–Smirnov test, for the different variables (i.e., team formation, player role, and ball-possession status) and their classes. For these variables, the players’ Voronoi cell areas presented statistical differences, which were sensitive to team formation classes (i.e., defenders, midfielders, and forwards) and relative pitch location (interior or exterior in the effective play space). Differences were also found between players with similar roles when in different team formations. Our results showed that team formation and players’ roles constrain their interpersonal linkages, resulting in different spatial dominance patterns. Using positional data captured by optical sensors, Voronoi diagrams can be computed into compound variables, which are meaningful for understanding the match and thus offer information to the design representative training tasks.

## 1. Introduction

In recent years, the technological progress around spatial location systems and positional data has had a growing impact on our societies and in all investigation fields [1,2], including sport sciences [3] and high-performance football [4].

This increase in the volume of data [5] can better inform coaches about the performance of their teams, including tactical behaviour [6]. Importantly, teams’ performance is based on the coordinated decisions of their players [7], which form team synergies (spatial-temporal patterns of coordination) guided by shared affordances [8].

Affordances are properties of the environment that relate to the individual characteristics, implemented by specific perception–action cycles, i.e., “action specific relations that exist between the skills/capacities of an individual performer and the action relevant properties of a [perceived environmental] task” ([8] p. 4). Training develops football players to become attuned to affordances of the match, namely, those constrained by match phases such as ball possession status [9,10]. Such *attunement* is better developed if coaches pursue the *representativeness* of their training exercises [11] through the manipulations of relevant task constraints [12].

When training for a match, coaches constrain the emergence of the affordances perceived by players and, consequently, their interactions with teammates and opponents [13]. For this purpose, there are evidence-based match-space criteria for training design. For example, coaches can define the space of their training exercises from generic benchmarks such as the *Game Intensity Index* (GII) [14]. The GII establishes a parallelism of the training surface in terms of square meters per player with that of competition. However, this is a very broad reference, which, in high-competition football, is equivalent to an area of 325 m^2^ per player (68 m × 105 m/22 *players*). It is no surprise that studies with *small-sided and conditioned games* (SSCG) suggest the use of *relative space per player* (RSP). The RSP corresponds to an area per player that derives from the smallest rectangle where all field players fit [15]. Similarly, Silva and colleagues [16] divided the *effective play space* (EPS), which is the polygon of the smallest convex hull, by the number of players. Both RSP proposals have the merit of measuring what occurs in game spaces in training and competition. However, they do not consider the space outside the EPS and, consequently, the impact of team formation on players’ and teams’ metrics.

During a football game, players do not move randomly throughout the space [17]. Players’ movements and team coordination [18] are constrained by strategy [7], including the game system or *team formation* [19]. These formations constrain the spatial organisation of players in a team [20] and, thus, how they can form synergies [18]. Team formations are especially relevant to understand *interpersonal linkages* as “the specific contribution of each element to a group task” ([8] p. 8). Team formations are typically represented via a set of three or four numbers that indicate the number of players in each line (or sector) and express how the team is organised on the pitch. For example, “3-5-2” expresses that the team formation is composed of three defenders, five midfielders, and two attackers [20]. Moreover, each player in his/her sector has a specific spatial *role* [21,22] or playing position [21], which is tagged with a specific designation. Usually, it describes the player’s main role, considering both the sector to which they belong (e.g., defenders—all outfield players that are more implicated in defensive tasks) and information about their corridor and side (e.g., right lateral defender or left centre midfielder). Currently, in high-performance football, 3-5-2, 3-4-1-2, and 4-2-3-1 are among the most commonly used tactical team formations [6,23].

Team formation affects performance by, for example, influencing key performance indicators (KPIs) such as the *Effective Play Space* (EPS) or *Team Separateness* [22]. Although the clear influence of team formations and players’ role in individual and team performance, its relevance for understanding the synergies that emerge from players interaction in competition are still unclear.

Aiming to bridge this gap, we argue that if the players’ roles within a team formation influence team synergies, then it will be possible to identify their specific contributions. Nowadays, we can compute positional data obtained by different types of sensors (e.g., optical tracking, GPS, or RFID) and calculate team spatial–temporal patterns such as *spatial heatmaps* [24], *major ranges* [25], or *Voronoi diagrams* (VD). VD in particular assess players’ *space dominance* [26], as well as, at the team synergetic level, their interpersonal linkages ([8] p. 8), (e.g., maintain ball possession).

This paper aims to understand the influence of team formation and players’ roles in the players’ spatial dominance resulting from the dynamic interaction (interpersonal linkages) of both teams. Therefore, we expect the following:

- Interpersonal linkages among players are expressed by their spatial interactions and are constrained by team formation and players’ roles.
- Players’ spatial dominance could be operationalised by Voronoi diagrams (and related spatial statistics), which could capture differences according to team formations, players’ roles, and ball-possession status.

## 2. Materials and Methods

### 2.1. Data Sources

The data used in this paper were provided by STATS© and obtained through their systems of semi-automatic tracking [27] in ten *Ligue 1* matches (France) of the 2019–2020 season. Data were composed of players’ positional data (longitudinal and lateral coordinates) sampled at 10 Hz, and notational data describing match events (representing players’ contacts with the ball) and possession episode (PE) information (initial and final instants, team with ball possession).

### 2.2. Data Processing

The raw data were processed before analysis using the following procedures:

- For each match, the determination of team formations was performed in two steps:
  1. Using the STATS Edge Analysis application:
    - The match time was divided into six periods of 15 minutes, as suggested by Duarte and colleagues [28]; each period was subdivided in case there was a substitution.
    - The average longitudinal and lateral position of each player was computed throughout each time period.
  2. From these results and following the suggestion of Carling [29] and Bradley and colleagues [30], a panel of experts identified both team formations during each of the match intervals. The panel was composed of five coaches with at least ten years of professional experience at the highest level and holding an UEFA PRO certification.
- Team formation, players’ roles, and ball-possession status were considered crucial to data analysis in this paper; consequently, matches and periods within the matches were grouped and selected according to the following criteria:
  - For each match, there was an analysed team and an opponent team. For all matches and time periods, the opponent team was always the same and organised under the same team formation (3-5-2). The analysed team was always a different one, forming two groups of five matches. In one group, the analysed team played mostly in a 4-2-3-1 team formation, and in the other group mostly with a 3-4-1-2 team formations.
  - Within each match, only periods in which teams maintained their team formation (4-2-3-1 or 3-4-1-2 for the analysed team and 3-5-2 for the opponent team) were used. All other periods, either where teams played with different formations or where they were not complete (e.g., after a red card), were discarded.
- Match periods were further filtered so that only open plays were considered; i.e., set plays and time gaps without play were discarded. Each open play was subdivided into ball-possession episodes (PEs). Each PE starts at the instant when a team recovers the ball and ends when that team loses control of the ball. According to STATS© reference manual [31], at least two consecutive events were necessary to form a PE. Each PE was classified, given the analysed team’s ball possession status, as *in possession* or *out of possession*. The 4-2-3-1 formation comprised 999 possession episodes (499 in possession, 500 out of possession), whilst the 4-2-3-1 formation comprised 1199 possession episodes (601 in possession, 598 out of possession).
- The role of each player of the analysed team was classified according to his spatial average position in the respective team formation. Table 1, adapted from Riezebos [21], identifies these roles for the two team formations: 4-2-3-1 and 3-4-1-2.
- The average value of the Voronoi cell area (VA) during the PE was computed for each player of the analysed team. VAs are computed at each time frame, using the procedures described by Kim [32], considering all the players of both teams.

**Figure 1:**
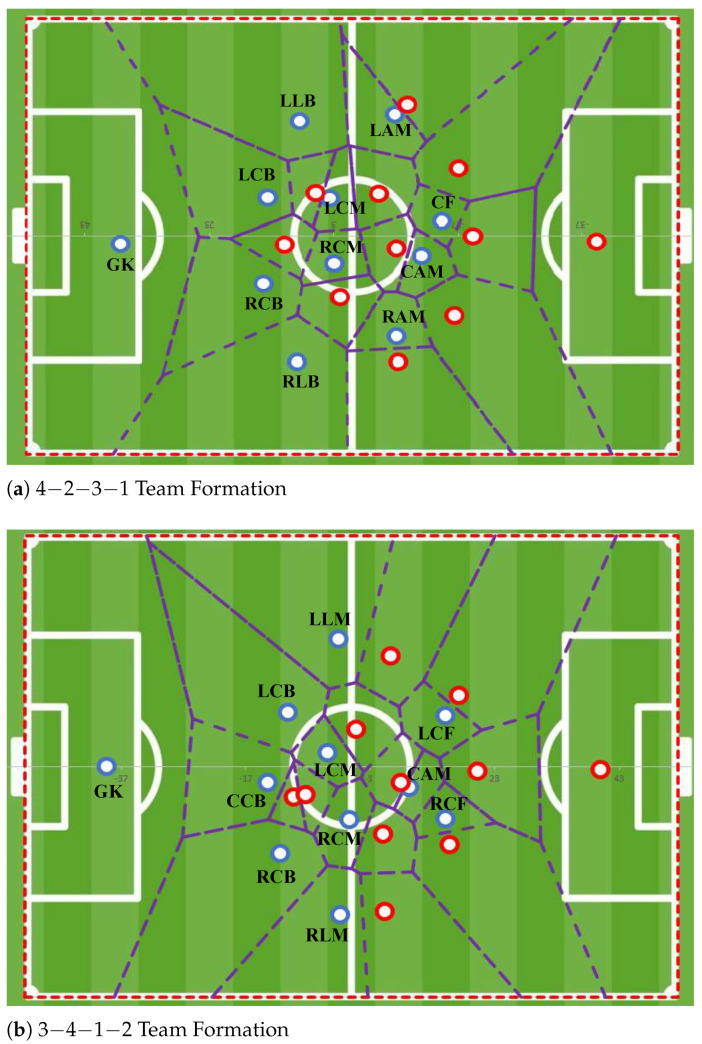
Voronoi diagrams for analysed teams (blue) in two team formations ((**a**) 4-2-3-1 and (**b**) 3-4-1-2) and the opposing team (red).

Figure 1 illustrates the Voronoi diagrams (VDs) obtained from players’ roles with different analysed team formations. Although VDs are computed at each time frame, in Figure 1, each player is represented at the average position along the longitudinal and lateral axes for the five matches considered, and their Voronoi area is circumscribed by dashed lines. Players from the analysed teams (in blue) are indicated using their role tag.

The VDs in Figure 1 exemplify the influence of the opponent team in the VA of the analysed team players, thus capturing the interaction of all players on the pitch and not only with his teammates [33]. This interaction is crucial for the relative position of a given player in the effective play space (EPS). For example, the *Right Centre Forward* (RCF) and the *Left Centre Forward* (LCF) in the 3-4-1-2 team formation, despite being the most forward elements of their team, occupy interior areas in the game space due to the interaction with their opponents.

### 2.3. Statistical Analysis Methods

Statistical analysis was performed by computing and comparing the probability density functions of the players’ mean Voronoi areas (VAs) during each possession episode (PE). Probability density functions are represented as violin plots using kernel smoothing and compared using the Kolmogorov–Smirnov statistic. For the Kolmogorov–Smirnov test, “H0: same distribution” is used as the null hypothesis, with a significance level set at 0.05 (i.e., the alternative hypothesis is assumed if p<0.05).

## 3. Results

### 3.1. Comparing Players’ Voronoi Areas (VA) within the Same Team Formation (TF)

**Figure 2:**
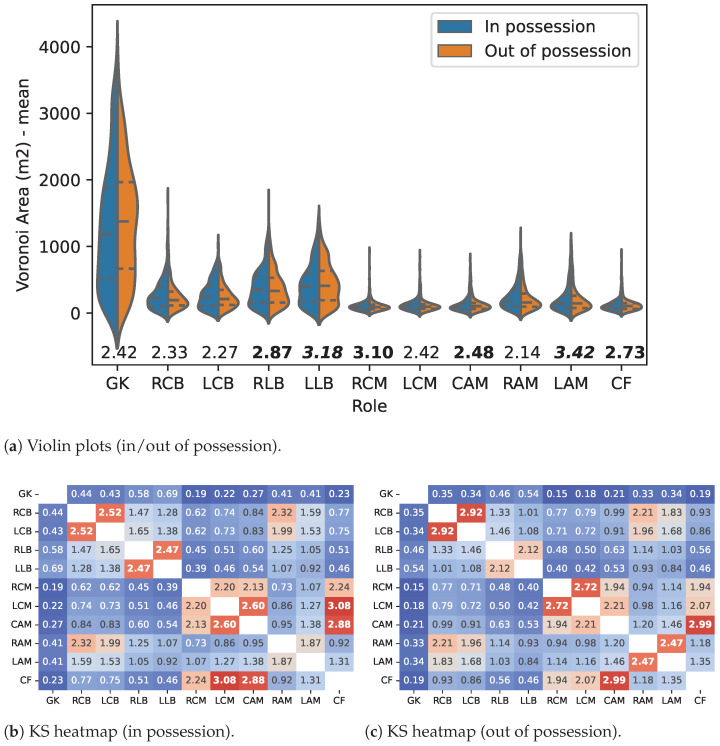
Players’ Voronoi area probability density function in the 4-2-3-1 team formation. *Note:* Violin plots (**a**) and heatmaps (**b**,**c**) comparing players’ Voronoi area probability density function (less similar in blue, more similar in red). (The differences that are statistically not relevant are highlighted in **bold**).

The results of comparing players’ *Voronoi Areas* (VA), according to their role and ball possession status (in possession and out of possession) within the 4-2-3-1 *team formation* (TF), are presented in the violin plots (a) and heatmaps (b and c) of Figure 2. The values in Figure 2 correspond to the Kolmogorov–Smirnov statistic values quantifying the differences between VAs and their statistical significance.

In Figure 2, the value indicated for each role *i* was computed as $V_{KS}\left(i\right)=-log\left(KS\left(P_{i},Q_{i}\right)\right)$ where KS is the Kolmogorov–Smirnov statistic and $P_{i}$,$Q_{i}$ are the VA probability density functions for a player with role *i* when the analysed team is *in* and *out* of ball possession, respectively. Differences between ball possession status were statistically significant (p<0.05) except for roles highlighted in bold.

The differences between VAs for all possible role pairs are represented in the heatmaps of Figure 2. The value indicated in cell i,j was computed as $V_{KS}\left(i,j\right)=-log\left(KS\left(P_{i},Q_{j}\right)\right)$ where KS is the Kolmogorov–Smirnov statistic and $P_{i},Q_{j}$ are, respectively, the VA probability density functions of players with role *i* and *j*. Differences between role pairs were statistically significant (p<0.05) except for pairs highlighted in bold.

Figure 2 clearly expose the differences in the distribution of players’ VA, according to their role and ball possession. Apart from the Goalkeeper’s (GK) specific case, the violin plots also differentiate players’ roles according to their sector (back vs. forward) and to their relative location (interior vs. exterior) in the *Effective Play Space* (EPS). Despite the general trend to find significant differences in the VA of players with different roles, ball-possession status also has an impact on VA similarity, mainly in the cases where differences were non-significant.

When the analysed teams were in possession of the ball, the non-significant differences were observed between players of the same sector, namely, between central backs (RCB and LCB), lateral defenders (RLB and LLB), and midfielders (CAM and LCM), and also between the Centre Forward (CF) and two midfield interior players (CAM and LCM). On the other hand, when the analysed teams were out of ball possession, non-significant differences remained between RCB and LCB and between CAM and CF. Except for the new non-significant differences between the defensive midfielders (RCM and LCM) and between wingers (RAM and LAM), all the others were now statistically significant.

**Figure 3:**
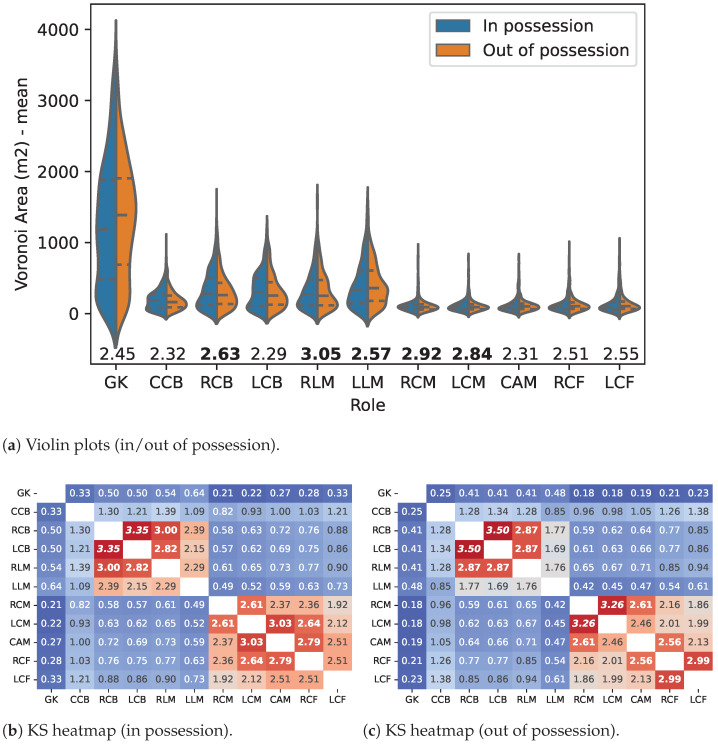
Players’ Voronoi area probability density function in the 3-4-1-2 team formation. (*Note:* Violin plots (**a**) and heatmaps (**b**,**c**) comparing players’ Voronoi area probability density function (less similar in blue, more similar in red). The differences that are statistically not relevant are highlighted in **bold**).

The same process was applied to the 3-4-1-2 team formation (TF). Players’ VA distribution is presented in the violin plots and their Kolmogorov-Smirnov statistics heatmaps in Figure 3.

Similarly, to the 4-2-3-1 team formation, smaller VAs were found for players who usually play in the interior regions of the EPS (RCM, LCM, CAM, RCF, and LCF). In addition, the third central back (CCB) seems to have even smaller areas than the players of the first defensive line (RCB and LCB).

Once more, the ball-possession status significantly influenced only some of the roles (GK, CCB, LCB, CAM, RCF, and LCF). All the other roles presented non-significant differences (highlighted in bold).

In this TF, VA distribution continues to generally allow the differentiation between players’ roles. However, the number of non-significant differences increases, showing a higher similarity between the VA of different roles, e.g., in the defensive line (CCB being the exception). The VA differences between role pairs were statistically significant (p<0.05), with the following exceptions (highlighted in bold in Figure 3):

1. In possession: RCB–LCB; RCB–RLM; LCB–RLM; RCM –LCM; LCM–CAM; LCM–RCF; CAM–RCF;
2. Out of possession: RCB–LCB; RCB–RLM; LCB–RLM; RCM–LCM; RCM–CAM; RCF–CAM; RCF–LCF.

### 3.2. Comparing Players’ Voronoi Areas (VA) between Different Team Formations (TF)

**Figure 4:**
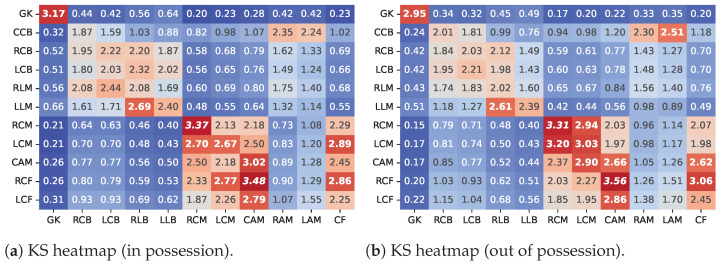
Comparison of the VA of players’ roles in both team formations.

Finally, the distribution of VA was compared according to players’ roles between team formations (Figure 4).

Both comparisons reveal a natural similarity between players in the same sector. However, the degree of similarity is not equal across sectors and shows important differences between defenders, midfielders, and attackers. In fact, in the defensive sector, all central backs showed significant differences between the TFs. In the lateral backs, the only non-significant difference was found between the LB and the RM. In the lateral backs, the only non-significant difference was found between the LB and the RM.

Concerning the midfielders, distinct analyses should be made for the interior and exterior players. In fact, several non-significant differences were found between the two TFs regarding the interior roles, showing that, for this sub-group, the choice between the 4-2-3-1 and 3-4-1-2 formation did not have a big influence on players’ VA, regardless of the team’s ball-possession status.

However, for the wingers (RAM and LAM) of the 4-2-3-1 formation, a clear different spatial pattern was found in these players’ VA distribution, which does not resemble any other role in the 3-4-1-2 TF (with the exception of the CCB).

Finally, for the attackers, several non-significant differences were found in the comparisons of the central forwards’ VA. These are found between players of the attacking sector from the two TFs and between players of the attacking and midfield sectors. This was especially evident in the comparisons with the attacking midfielders (CAM).

## 4. Discussion

The aim of this study was to understand the influence of *team formation* (TF) and players’ roles in their dynamic interaction (interpersonal linkages). For this purpose, *Voronoi diagrams* (VD) were used to assess the differences in players’ spatial dominance resulting from their interactions according to ball-possession status in high-performance football teams.

The observed results support some important reflections on the division of space, according to TF, players’ roles, and the ball-possession status. When analysing the spatial patterns within the same TF, the differences between players’ VA were generally found to be statistically significant according to their roles.

The results from Fonseca and colleagues [9], showing the influence of ball-possession status on players’ *Voronoi cell areas* (VA), do not universally apply but are dependent on *players’ roles*. With few some exceptions, these differences demonstrate the existence of different affordances according to players’ roles, especially between *sectors* and according to the relative location (*interior* or *exterior*) of each player in the *Effective Play Space* (EPS). The VA of each player role varies according to these two general criteria, influencing players’ interpersonal linkages in the establishment of collective synergies.

Resulting from the teams’ interactions and due to the nature of the VA metric, players who usually act in the interior of the EPS (midfielders and centre forwards) had smaller areas; players who play in the periphery of the EPS had larger VAs; and the VAs of the wingers or attacking midfielders (RAM and LAM) were intermediate (possibly because they alternate between interior and exterior spaces in the EPS). Additionally, more defensive players (who occupy positions closer to their own goal) generally deal with wider VAs, while the most offensive players usually deal with smaller VAs.

Although some role pairs show a certain degree of symmetry (RCB–LCB or RCM–LCM), this is not found globally. This is the case for the attacking midfielders (AMR–AML) in the 4-2-3-1 TF or the players of the lateral corridors (RM–LM) in the 3-4-1-2 TF. In comparing the two TFs analysed, it is important to note how VA patterns significantly change across the players of the defensive sector, exposing differences in the spatial affordances when a team plays with a first defensive line with three or four players. However, other roles were very similar in both team formations. Apart from the expected case of the GK, this similarity was especially true in the interior (centre) midfield positions. Our results also underpin some practical clues that we find relevant to the football coaches’ work. First is the need to consider TF and players’ roles as important constraints during the design of training exercises [19]. This implies the need to manipulate and measure the space per player role that actually occurs within these drills in reference to the competitive patterns [18].

Additionally, the fact that no significant differences were found between some players’ roles (e.g., between the two central backs or the two more defensive midfielders—a fact found in both TFs and independent of ball possession status) may indicate that players can eventually switch more easily between these roles. This is particularly relevant in situations out of the game plan, e.g., when replacing an injured player.

However, as most role-pair comparisons presented significant differences, coaches need to be aware of the difficulty for players to adapt to the spatial affordances associated with different roles. Even within the same sector, switching sides may imply different spatial affordances due to the non-symmetry detected in some roles. The difference between players’ role patterns that were expected to be similar between the two TFs highlights the need for coaches to dedicate enough training time to attune players, individually and collectively, to the spatial affordances that emerge from the strategical option for a given TF [34]. Coaches should be aware that a sudden change in TF may cause more difficulties in adapting to their players than to their opponents. Moreover, differences in VA spatial patterns, according to the TF and players’ roles, may also imply the need to properly consider them in the long-term training processes of youth footballers. For example, by introducing a certain degree of variability in the role, coaches can avoid a possible early specialisation [35].

Voronoi diagrams can thus be considered a useful tool to study teams in competitions (match analysis) and as an auxiliary metric to the design of representative training exercises. After characterising the VA of each player role in a given team organisation (TF) during competition, the next step is to use the same tools in training exercises. By measuring players’ VA in each exercise, it will be possible to compare the data obtained in the context of training with the values of the respective team in the context of competition. This can constitute a possible way to quantify, in spatio-temporal terms, their representativeness degree, with more detail than with *Game Intensity Index* [14] or the *Relative space per player* [15,16]. In particular, and contrary to the relatively simplistic idea proposed in [36] that 320 m^2^ per player would be more representative to design Small-Sided and Conditioned Games (SSCG), VD can help coaches to more effectively manipulate training surfaces. In fact, the adoption of VD to assess players’ spatial patterns can help in the definition of more suitable dimensions for each training exercise, adjusting them to the global TFs and players’ roles. The use of VD-based tools can contribute to achieving a higher degree of representativeness of training exercises, in both SSCG and large-sided games [37].

The differences between the spatial patterns of players’ roles within the same TF also underline the importance of coaches designing *supraspecific* training tasks, i.e., the specific training that goes beyond simply training with the ball [38]. *Supraspecificity* implies the design of tasks that are based not only on the football’s general dynamics but also on the specific constraints of each team (e.g., coach’s game model, including team formations), which has an important role in the development of the interpersonal linkages and collective synergies that underpin team performance [8].

## 5. Conclusions

This study exposes how *team formations* and *players’ roles* influence the spatial patterns of their Voronoi cell areas. It underlines the importance of considering these features when coaches design training exercises, as they constrain players’ interpersonal linkages in the establishment of team synergies and collective performance. Consequently, this study reinforces the need to train *ecologically* [39], as a pathway for players’ progressive attunement to the affordances of the competitive environment, i.e., through *representative training* [11]. We believe that the assessed methods and their results can contribute to leveraging the utility of optical tracking systems in sports and ultimately to the tactical performance of high-level football teams. 

## Figures and Tables

**Table 1:** Player’s role in 4-2-3-1 and 3-4-1-2 team formations.

4-2-3-1		3-4-1-2	
***Tag***	***Description***	***Tag***	***Description***
GK	Goalkeeper	GK	Goalkeeper
LLB	Left Lateral Back	CCB	Centre Central Back
LCB	Left Central Back	LCB	Left Central Back
RCB	Right Central Back	RCB	Right Central Back
RLB	Right Lateral Back	LLM	Left Lateral Midfielder
LCM	Left Centre Midfielder	LCM	Left Centre Midfielder
RCM	Right Centre Midfielder	RCM	Right Centre Midfielder
LAM	Left Attacking Midfielder	RLM	Right Lateral Midfielder
CAM	Centre Attacking Midfielder	CAM	Centre Attacking Midfielder
RAM	Right Attacking Midfielder	LCF	Left Centre Forward
CF	Centre Forward	RCF	Right Centre Forward

## Data Availability

Third party data. Restrictions apply to the availability of these data. Data was obtained from STATS© company and are available from STATS© with their permission.

## Abbreviations

The following abbreviations are used in this manuscript:

CLA	Constraints-Led Approach
EPS	Effective Play Space
GII	Game Intensity Index
KS	Kolmogrov-Smirnov
PE	Possession Episodes
RSP	Relative Space per Player
SSCG	Small-Sided and Conditioned Games
TF	Team Formation
VA	Voronoi Area
VD	Voronoi Diagrams
*Note:*	Players’ role in 4-2-3-1 and 3-4-1-2 team formations are defined in Table 1.
